# Detecting short-term post-traumatic stress disorder symptom increases among veterans: machine-learning analysis integrating wearable sensor and daily self-report data

**DOI:** 10.1192/bjo.2026.12055

**Published:** 2026-09-01

**Authors:** Daniel Leightley, John Prindle, Shaddy Saba, Bistra Dilkina, Eric R. Pedersen, Emily Dworkin, Esther Howe, Praneeth Thota, Sriram Nuthi, Angeles Sedano, Jordan P. Davis

**Affiliations:** School of Life Course & Population Sciences, King’s College London, UK; Suzanne Dworak-Peck School of Social Work, University of Southern California, USA; Silver School of Social Work, New York University, USA; Viterbi School of Engineering, University of Southern California, USA; Keck School of Medicine, University of Southern California, USA; Department of Psychiatry and Behavioral Sciences, University of Washington, USA; Department of Psychology, University of California Berkeley, USA; RAND Corporation, Santa Monica, California, USA

**Keywords:** Cannabis, PTSD, veteran, machine learning methods, mental health services

## Abstract

**Background:**

Post-traumatic stress disorder (PTSD) symptoms can fluctuate substantially over short periods, yet routine screening typically relies on infrequent self-report. Wearable sensors provide continuous behavioural and physiological signals that may help identify periods of elevated risk.

**Aims:**

This study aimed to evaluate whether combining wearable sensor features with daily self-report data could identify short-term PTSD symptom increases among recently discharged veterans.

**Method:**

Seventy-four veterans wore commercial activity trackers and completed brief daily questionnaires over 87 days. For each participant, we defined an individual baseline by using the first 14 days of PTSD scores. Wearable variables were transformed into baseline-referenced deviation features to capture departures from personal norms. Missing data were addressed with multiple imputation by chained equations. Candidate predictors were prioritised with least absolute shrinkage and selection operator regression, and a set of machine-learning classifiers was evaluated. Primary performance was assessed by using the area under the precision–recall curve (PR AUC).

**Results:**

Across feature set sizes (*k* = 1–25), performance peaked at *k* = 17. At this iteration, LightGBM achieved the strongest discrimination (PR AUC 0.86 (s.d. 0.07); area under the receiver-operating characteristic curve 0.89 (s.d. 0.04)) with a precision of 0.67 (s.d. 0.08), recall of 0.64 (s.d. 0.08) and F1 of 0.65 (s.d. 0.07). Key predictors reflected a multimodal profile, combining self-reported affect and perceived stress with wearable indicators of sleep continuity, activity variability and autonomic regulation.

**Conclusions:**

Baseline-referenced wearable features combined with daily self-report may help identify near-term PTSD symptom increases among recently discharged veterans with elevated PTSD symptoms and problematic cannabis use. Future work should validate performance in broader PTSD populations, including samples without problematic cannabis use.

Post-traumatic stress disorder (PTSD) is defined in the DSM-5 as a trauma-related condition characterised by intrusive symptoms, avoidance, negative alterations in cognition and mood, and hyperarousal, persisting for more than a month and associated with functional impairment.^1,2^ Early identification of PTSD, and of symptom escalation among those with existing difficulties, is important to reduce chronicity and support timely access to care.^3,4^ Among veterans in the USA, recent national estimates place lifetime PTSD at between 9 and 10%, and past-month PTSD is around 5%.^5^ Estimates vary, reflecting differences in sampling and measurement, and the absence of a central register of PTSD diagnoses. Despite the need for support, many active duty military personnel and veterans do not seek help because of stigma, concerns about career impact and logistical barriers.^6,7^

Traditional screening relies on self-report questionnaires and clinical interviews. These approaches may miss short-term symptom fluctuations and can be affected by recall bias, limiting the timeliness and precision of risk detection.^8,9^ Intensive longitudinal data could address some of these limitations by sampling experiences in real time in daily life, thus improving ecological validity.^10,11^ Digital phenotyping extends this further through the moment-by-moment measurement of behaviour and physiology in everyday life, using data collected from smartphones and wearable devices. It can provide continuous measures, allowing for the detection of changes that precede symptom escalation and to deliver just-in-time support without relying solely on participant self-report.^11^

Physiological and behavioural signals measured by wearable devices have been shown to map onto core PTSD symptoms.^12^ PTSD has been associated with reduced resting heart rate variability and broader autonomic dysregulation, and sleep disturbance (e.g. insomnia and nightmares) is highly prevalent and clinically meaningful in this population.^13,14^ Together, these findings suggest that multimodal sensing may help detect near-term changes in risk. Initial analyses from our feasibility study (MAVERICK study) support this. In 74 recently discharged veterans (within 3 years of service), poorer sleep quality predicted higher next-day stress and subsequent alcohol consumption, and PTSD symptoms were strongly correlated with daily negative affect.^8,9^ This work focused on recently discharged veterans with elevated PTSD symptoms and problematic cannabis use because this group represents a clinically important and potentially high-need subgroup. The transition out of military service can be associated with changes in routine, social support, sleep and substance use, and co-occurring cannabis use may interact with PTSD symptoms, sleep and physiological regulation.

Machine-learning techniques are being applied to improve PTSD detection,^15,16^ and can use complex patterns in multimodal data to identify risk with promising precision. For example, one study applied machine learning to audio and video features derived from clinical interviews (facial expressions, speech and language content) and detected PTSD in trauma patients with around 83% accuracy.^17^ In our initial pilot study, supervised learning was used to predict clinically significant daily PTSD symptom increases in veterans; daily negative affect was the strongest predictor, with depressed mood and perceived stress also retained, and co-occurring high negative affect and anxiety corresponding to an >55% probability of symptom escalation.^18^ This diary-based evidence provides proof of concept that short-term symptom increases can be predicted from high-frequency measurements, motivating efforts to extend prediction not just by using self-report, but also passive digital phenotyping. Passive data from smartphones and wearables can capture behavioural and physiological indicators between scheduled assessments in everyday life, which machine-learning algorithms may analyse for short-term detection of symptom escalation.^19^ This can also be used to personalise care through the use of push notifications as part of a behaviour change framework.^20^

Two practical considerations are central to this approach. First, there is substantial between-person heterogeneity in typical physiology and behaviour, meaning that deviations from an individual’s own baseline are likely to be more informative than absolute levels. Second, passive sensing and daily assessments are often incomplete (e.g. device non-wear, syncing failures and skipped questionnaires), and missingness must be handled carefully to avoid biased or unstable models.

Therefore, in this study, we use data from the MAVERICK feasibility study to evaluate whether machine-learning models can classify short-term, within-person PTSD symptom increases, using a combined feature set of baseline-adjusted wearable indicators and daily self-report measures among recently discharged veterans with elevated PTSD symptoms and problematic cannabis use. Specifically, we aimed to (a) derive baseline-adjusted wearable features that quantify departures from each participant’s typical patterns; (b) address missing data with a principled imputation strategy; (c) identify the most informative predictors across modalities; (d) train and compare multiple classifiers with cross-validation and (e) characterise the features most strongly associated with probable PTSD symptom escalation.

## Method

The authors assert that all procedures contributing to this work comply with the ethical standards of the relevant national and institutional committees on human experimentation and with the Helsinki Declaration of 1975, as revised in 2013. All procedures in the MAVERICK study^8,9,18,21^ involving human patients were approved by University of Southern California Institutional Review Board (identifier #UP-20-00853). All data were stored securely, deidentified before analysis and handled in accordance with institutional policies. Participants provided written informed consent before enrolment, with specific content for wearable data collection.

### Study design and participants

Active and passive data were collected via the MAVERICK smartphone app, a Research Viable Product,^22^ developed by the MAVERICK research team through a co-design process with veterans and stakeholders, with privacy-by-design principles embedded throughout development.^22,23^ The app delivered brief daily questionnaires and linked to Apple Health, Google Fit and Garmin for passive sensor collection.

MAVERICK recruited 74 USA veterans who had been discharged from the USA Armed Forces within the previous 3 years, had elevated PTSD symptoms and reported problematic cannabis use. Participants were recruited through social media and a recruitment agency.^21^ Participants were asked to wear a FitBit or another compatible wearable device for 87 days (including day and night). They were also asked to complete a baseline screening survey, daily diary surveys throughout the monitoring period and follow-up assessments at 28-day intervals. Participants received compensation for each completed assessment.

Baseline characteristics are summarised in Table 1 (*n* = 74; mean age 34.2 years; 18.9% female; racially/ethnically diverse). On average, participants reported 2.5 military deployments; employment ranged from full time to unemployed and retired. Mean baseline PTSD and depression scores were 39.0 (s.d. 16.3) and 10.4 (s.d. 5.6), respectively. Mean days of use in the past 30 days were 20.4 for cannabis and 8.7 for alcohol, with 3.4 binge-drinking days; 42.1% did not have their own wearable device and received a FitBit from the study, and 76.1% owned an Apple smartphone.

**Table 1:** Baseline demographics Table 1 long description.

Demographic	Mean [s.d.] / *n* (%)
Age, years	34.2 [8.01]
Female	14 (18.9)
Race/ethnicity	
White	34 (45.9)
Hispanic	27 (36.5)
Black	7 (9.5)
Asian	2 (2.7)
American Indian/Alaska Native	1 (1.4)
Other/multi-race	18 (24.3)
Number of deployments	2.49 [2.91]
Employment	
Part time	20 (27.0)
Full time	25 (33.8)
Unemployed	17 (23.0)
Retired	12 (16.2)
Post-traumatic stress disorder	39.0 [16.3]
Depression	10.4 [5.66]
Substance use	
Cannabis (days/month)	20.4 [11.1]
Cannabis consequences	3.23 [3.63]
Alcohol (days/month)	8.71 [8.18]
Binge (days/month)	3.38 [4.84]
Study provided watch (FitBit)	32 [42.1]
Own an Apple smartphone	54 [76.1]

### Data collection

A brief summary of the data collection approach is presented hereafter; for a full overview, see Leightley et al.^21^

#### Active data collection

Two daily questionnaires (morning and evening) assessed PTSD symptoms, positive and negative affect, perceived stress, prior-night sleep quality, pain and substance use behaviours (hours high from cannabis since the previous entry, number of alcoholic drinks since the previous entry). At baseline, we recorded demographics, service history and standardised psychosocial scales, including measures of depressive and anxiety symptoms, insomnia, PTSD severity, loneliness, pain, impulsivity (total and subscales), cannabis-related motives and consequences, perceived stress, cognitive/threat appraisal, alcohol use patterns (including solitary and binge use), cannabis use patterns and a traumatic brain injury screen. Most questionnaire items used Likert-type response options and were aggregated to the calendar day per participant. To assess PTSD severity at baseline, the 20-item PTSD Checklist for DSM-5 (PCL-5^24^) was used. Participants indicated the extent to which they were bothered by specific PTSD symptoms in the past month, from not at all (0) to extremely (4). A four-item, short-form version of the PCL-5^25^ was used to assess daily PTSD symptoms. Further details on measures collected are reported elsewhere.^8,9,21^

#### Passive data collection

Wearable data streams covered six domains: (a) physical activity metrics (e.g. active minutes, energy expenditure), (b) distance travelled, (c) heart rate, (d) respiratory rate, (e) sleep duration and quality, and (f) step counts (see Supplementary Appendix 1 for a full list). Participants were also able to record manually food logging events via Apple Health/Google Fit, which were also used in analysis. Data were time-stamped, linked to a unique participant identifier and stored separately from identifiable information.

### Preprocessing and imputation

#### Indexing

All records were aligned to a study-day index from day 1 to day 87. Variables with >70% missingness or with no observed variation (all-zero values) were removed. Remaining missing data were handled with multiple imputation by chained equations, generating 50 completed data-sets under a missing-at-random assumption. Imputation models were matched to variable type and included relevant covariates to preserve observed associations. Imputation was performed with the MICE package (version 3.19.0) in R for Mac (R Foundation for Statistical Computing, Vienna, Austria; https://cran.r-project.org/web/packages/mice/index.html).^26^

#### Wearable features

Wearable variables were harmonised across smartwatch device types to ensure consistent measurement definitions across Apple Health, Google Fit and Garmin sources. To account for between-person differences, we defined the first 14 monitoring days as an individual baseline window. For each wearable variable, we computed the participant-specific baseline mean and standard deviation. For subsequent time points (days >14), we derived three baseline-adjusted feature sets: (a) *z*-scores (value minus baseline mean, divided by baseline standard deviation, with a small constant added to avoid division by zero); (b) absolute deltas (value minus baseline mean) and (c) relative deltas (absolute delta divided by the absolute baseline mean). We additionally summarised post-baseline averages of these derived features for each participant and quantified instability as changes in variability relative to baseline, captured as differences in standard deviation and range.

#### Self-report measures

Daily self-reports were indexed by participant and index day and then merged with the wearable features at the participant-day level. The imputed self-report matrix comprised 39 candidate daily variables.

### Model evaluation outcome

We defined PTSD increase as a within-participant indicator of short-term symptom escalation. For each participant, data obtained between day 1 and 14 were used to establish an individual baseline distribution of daily PTSD scores. This operational definition reflects within-person symptom escalation rather than diagnostic change. For each subsequent day, the outcome was coded as 1 if the participant’s daily PTSD score was at least one baseline standard deviation above their baseline mean, and 0 otherwise. The outcome was derived after multiple imputation of daily self-report data and was intended as a conservative operational marker of short-term symptom escalation rather than a diagnostic label.

### Feature selection

Candidate predictors included daily self-report measures and engineered wearable features (baseline-standardised scores, absolute and relative deltas, and variability shifts). Selection was performed independently within each of the 50 imputed data-sets and then pooled. Before modelling, we applied a pre-imputation coverage filter (≥30% observed) to screen candidates. Within each imputed data-set, predictors were *z*-standardised, outcomes were aligned at the participant level and stratified cross-validation folds were limited by the minority-class size (maximum 10). Within each source domain (self-report, activity, distance, food, heart, respiratory, sleep, steps) we fitted the least absolute shrinkage and selection operator regression model with class-balanced weights.^27^ Regularisation strength was chosen by stratified out-of-fold log loss over a small grid. For each feature we recorded the absolute coefficient per imputation and derived a pooled ranking by combining selection frequency (non-zero coefficient across imputations) with a normalised mean-coefficient term. We then constructed nested feature sets of size *k* and repeated model training for *k = {1, 2, …, 25}*. For wearable domains, the top *k* features were taken within each domain to preserve sensor breadth; for self-report, the top *k* features were taken from the self-report pool.

### Machine-learning models and evaluation

In this exploratory analysis, models were trained on a participant-level matrix that always combined the selected wearable features with the selected self-report features, aligned by participant identifier. To minimise overfitting, we performed stratified ten-fold cross-validation and restricted all scalar preprocessing steps (median imputation of residual missing values and *z*-standardisation) to the training folds. We acknowledge that the initial feature ranking was derived on the full data-set, so performance estimates may be optimistic and therefore should be interpreted as exploratory.

We benchmarked a broad panel of classifiers used often in this type of evaluation: logistic regression (balanced), Support Vector Machine with radial basis function kernel (SVM RBF),^28^ Linear Support Vector classifier (SVM linear), Random Forest,^29^ Extremely Randomised Trees,^30^ Gradient Boosting, AdaBoost,^31^ Gaussian naïve Bayes, *k*-Nearest Neighbours, Decision Tree, Stochastic Gradient Descent classifier with log-loss and Ridge Classifier. We also evaluated XGBoost,^32^ LightGBM^33^ and CatBoost, using default binary probabilistic settings. Probability scores were obtained via ‘predict_proba’ function or via the decision function of Scikit Learn Python package.^34^

Evaluation used stratified ten-fold cross-validation with a fixed random seed. To minimise information leakage, all preprocessing steps were fitted within each training fold and then applied to the corresponding test fold, including median imputation of any residual missing predictor values followed by *z*-standardisation. Class imbalance was addressed with class-balanced loss where supported. For *k*-Nearest Neighbours, the number of neighbours was constrained within each fold to remain feasible given the training set size.

We computed, for each fold, accuracy, precision, recall, F1 score, area under the receiver-operating characteristic curve (ROC AUC) and area under the precision–recall curve (PR AUC). Given class imbalance, PR AUC was the primary metric used for model comparison, reflecting the ability to rank true cases above non-cases. For each classifier, performance metrics were pooled across folds and imputations and summarised as the mean and standard deviation, with models ranked primarily by mean PR AUC. For tree-based methods, we extracted fold-specific feature importance estimates from the first fold to support interpretation.

## Results

Seventy-four recently discharged USA veterans contributed daily self-reports and continuous wearable data over 87 days. All models were trained on the combined feature set comprising wearable features and self-report variables, with performance pooled over stratified ten-fold cross-validation folds and imputations.

### Model performance for classifying short-term PTSD symptom increases

At *k* = 17 (Table 2), LightGBM performed the highest, with the highest PR AUC (0.86 (s.d. 0.07)), high ROC AUC (0.89 (s.d. 0.04)) and balanced operating-point metrics (precision 0.67 (s.d. 0.08), recall 0.64 (s.d. 0.08), F1 0.65 (s.d. 0.07)). XGBoost was the next highest non-linear model by PR AUC (0.80 (s.d. 0.08); ROC AUC 0.85 (s.d. 0.05)), but had lower precision and recall. Among linear baselines, Ridge Classifier and logistic regression achieved comparable discrimination (PR AUC 0.76 (s.d. 0.10) and 0.72 (0.09), respectively; ROC AUC 0.86 (s.d. 0.06) and 0.87 (s.d. 0.05), respectively), with balanced F1 scores (0.45 (s.d. 0.08) and 0.43 (s.d. 0.08), respectively).

**Table 2:** Cross-validated performance by model at best iteration (*k* = 17), ranked by PR AUC performance Table 2 long description.

Classifier	PR AUC (s.d.)	ROC AUC (s.d.)	Precision (s.d.)	Recall (s.d.)	F1 (s.d.)
LightGBM	0.86 (0.07)	0.89 (0.04)	0.67 (0.08)	0.64 (0.08)	0.65 (0.07)
XGBoost	0.80 (0.08)	0.85 (0.05)	0.41 (0.09)	0.40 (0.09)	0.38 (0.08)
Random Forest	0.78 (0.10)	0.84 (0.06)	0.07 (0.04)	0.06 (0.05)	0.06 (0.04)
CatBoost	0.77 (0.09)	0.85 (0.06)	0.06 (0.04)	0.05 (0.05)	0.05 (0.04)
Ridge Classifier	0.76 (0.10)	0.86 (0.06)	0.46 (0.08)	0.60 (0.08)	0.45 (0.08)
Support Vector Machine Linear	0.73 (0.10)	0.84 (0.07)	0.39 (0.08)	0.49 (0.08)	0.36 (0.08)
AdaBoost	0.73 (0.09)	0.80 (0.06)	0.33 (0.09)	0.35 (0.09)	0.32 (0.08)
Logistic regression	0.72 (0.09)	0.87 (0.05)	0.42 (0.09)	0.54 (0.09)	0.43 (0.08)
Support Vector Machine RBF	0.69 (0.10)	0.78 (0.06)	0.49 (0.09)	0.35 (0.09)	0.39 (0.09)
Gradient Boosting	0.64 (0.10)	0.75 (0.06)	0.25 (0.10)	0.34 (0.09)	0.27 (0.10)
k-Nearest Neighbours, Decision Tree	0.60 (0.11)	0.72 (0.07)	0.07 (0.04)	0.06 (0.05)	0.05 (0.03)
Stochastic Gradient Descent Classifier	0.56 (0.11)	0.77 (0.07)	0.38 (0.08)	0.61 (0.09)	0.42 (0.09)
ExtraTrees	0.52 (0.09)	0.67 (0.05)	0.07 (0.04)	0.06 (0.04)	0.06 (0.04)
Decision Tree	0.36 (0.12)	0.64 (0.08)	0.24 (0.09)	0.40 (0.10)	0.29 (0.09)
Naïve Bayes	0.27 (0.13)	0.53 (0.08)	0.23 (0.10)	0.75 (0.11)	0.35 (0.10)

PR AUC, area under the precision–recall curve; ROC AUC, area under the receiver-operating characteristic curve.

### Feature-set optimisation and neighbouring model iterations

Table 3 outlines the trade-off around *k* = 17. The mean PR AUC across models was 0.64 (s.d. 0.27) at *k* = 16, rose to 0.67 (s.d. 0.06) at *k* = 17 – an absolute gain of 0.03 – and then fell to 0.61 (s.d. 0.29) at *k* = 18. The lower dispersion at *k* = 17 (s.d. 0.06 versus 0.27 at *k* = 16 and 0.29 *at k* = 18) suggests greater agreement among models at this setting.

**Table 3:** Neighbouring iterations (*k* − 1, *k*, *k* + 1): PR AUC across models Table 3 long description.

Iteration	PR AUC (s.d.)	ROC AUC (s.d.)	Precision (s.d.)	Recall (s.d.)	F1 (s.d.)
16	0.64 (0.27)	0.77 (0.23)	0.67 (0.09)	0.63 (0.11)	0.64 (0.10)
17	0.67 (0.06)	0.78 (0.06)	0.58 (0.08)	0.56 (0.07)	0.57 (0.07)
18	0.61 (0.29)	0.75 (0.24)	0.27 (0.29)	0.34 (0.32)	0.27 (0.27)

PR AUC, area under the precision–recall curve; ROC AUC, area under the receiver-operating characteristic curve.

### Performance stability across feature-set sizes

Aggregating performance across folds (see Table 4), LightGBM achieved the highest mean PR AUC at 0.73 (s.d. 0.13), with SVM linear at 0.70 (s.d. 0.07), logistic regression at 0.69 (s.d. 0.05), and Random Forest also at 0.69 (s.d. 0.06). For ROC AUC, logistic regression led at 0.82 (s.d. 0.04), closely followed by LightGBM and SVM linear at 0.81 (s.d. 0.11) and 0.81 (s.d. 0.07), respectively. For operating-point performance, logistic regression delivered the best balance of precision and recall, yielding the highest F1 of 0.45 (s.d. 0.08), with a precision of 0.44 (s.d. 0.09) and recall of 0.55 (s.d. 0.07). Although Naïve Bayes attained the highest recall at 0.72 (s.d. 0.16), this was offset by low precision (0.23 (s.d. 0.05)), resulting in a modest F1 of 0.33 (s.d. 0.04). Tree-based ensembles showed mixed behaviour: Random Forest combined solid discrimination (PR AUC 0.69; ROC AUC 0.78 (s.d. 0.04)) with a very low F1 of 0.08 (s.d. 0.19), and XGBoost displayed a similar pattern (PR AUC 0.65 (s.d. 0.10); F1 0.28 (s.d. 0.13)). Overall, simpler linear models (logistic regression and SVM linear) provided the most stable and well-balanced performance across folds, whereas LightGBM offered the strongest PR AUC, but with greater variability in operating-point metrics.

**Table 4:** Overall PR AUC across all iterations (*k* = 1–25), by model Table 4 long description.

Classifier	PR AUC (s.d.)	ROC AUC (s.d.)	Precision (s.d.)	Recall (s.d.)	F1 (s.d.)
LightGBM	0.73 (0.13)	0.81 (0.11)	0.44 (0.21)	0.43 (0.16)	0.42 (0.18)
Support Vector Machine linear	0.70 (0.07)	0.81 (0.07)	0.41 (0.06)	0.54 (0.06)	0.42 (0.05)
Logistic regression	0.69 (0.05)	0.82 (0.04)	0.44 (0.09)	0.55 (0.07)	0.45 (0.08)
Random Forest	0.69 (0.06)	0.78 (0.04)	0.08 (0.20)	0.08 (0.19)	0.08 (0.19)
Ridge Classifier	0.69 (0.09)	0.79 (0.08)	0.41 (0.11)	0.53 (0.16)	0.42 (0.12)
CatBoost	0.69 (0.06)	0.78 (0.05)	0.05 (0.17)	0.05 (0.16)	0.05 (0.17)
AdaBoost	0.66 (0.06)	0.74 (0.07)	0.33 (0.09)	0.34 (0.08)	0.32 (0.09)
XGBoost	0.65 (0.10)	0.75 (0.10)	0.31 (0.14)	0.29 (0.13)	0.28 (0.13)
Support Vector Machine radial basis function kernel	0.64 (0.06)	0.73 (0.05)	0.46 (0.10)	0.40 (0.15)	0.40 (0.12)
Gradient Boosting	0.58 (0.08)	0.69 (0.07)	0.27 (0.11)	0.31 (0.09)	0.28 (0.10)
Stochastic Gradient Descent Classifier	0.57 (0.04)	0.76 (0.05)	0.38 (0.05)	0.56 (0.07)	0.41 (0.05)
ExtraTrees	0.53 (0.05)	0.67 (0.06)	0.09 (0.13)	0.08 (0.13)	0.08 (0.13)
k-Nearest Neighbours, Decision Tree	0.52 (0.05)	0.71 (0.05)	0.05 (0.13)	0.04 (0.12)	0.04 (0.13)
Decision Tree	0.34 (0.05)	0.58 (0.04)	0.23 (0.07)	0.32 (0.06)	0.25 (0.06)
Naïve Bayes	0.32 (0.10)	0.53 (0.04)	0.23 (0.05)	0.72 (0.16)	0.33 (0.04)

PR AUC, area under the precision–recall curve; ROC AUC, area under the receiver-operating characteristic curve.

### Predictor composition and multimodal feature profile

**Fig. 1:**
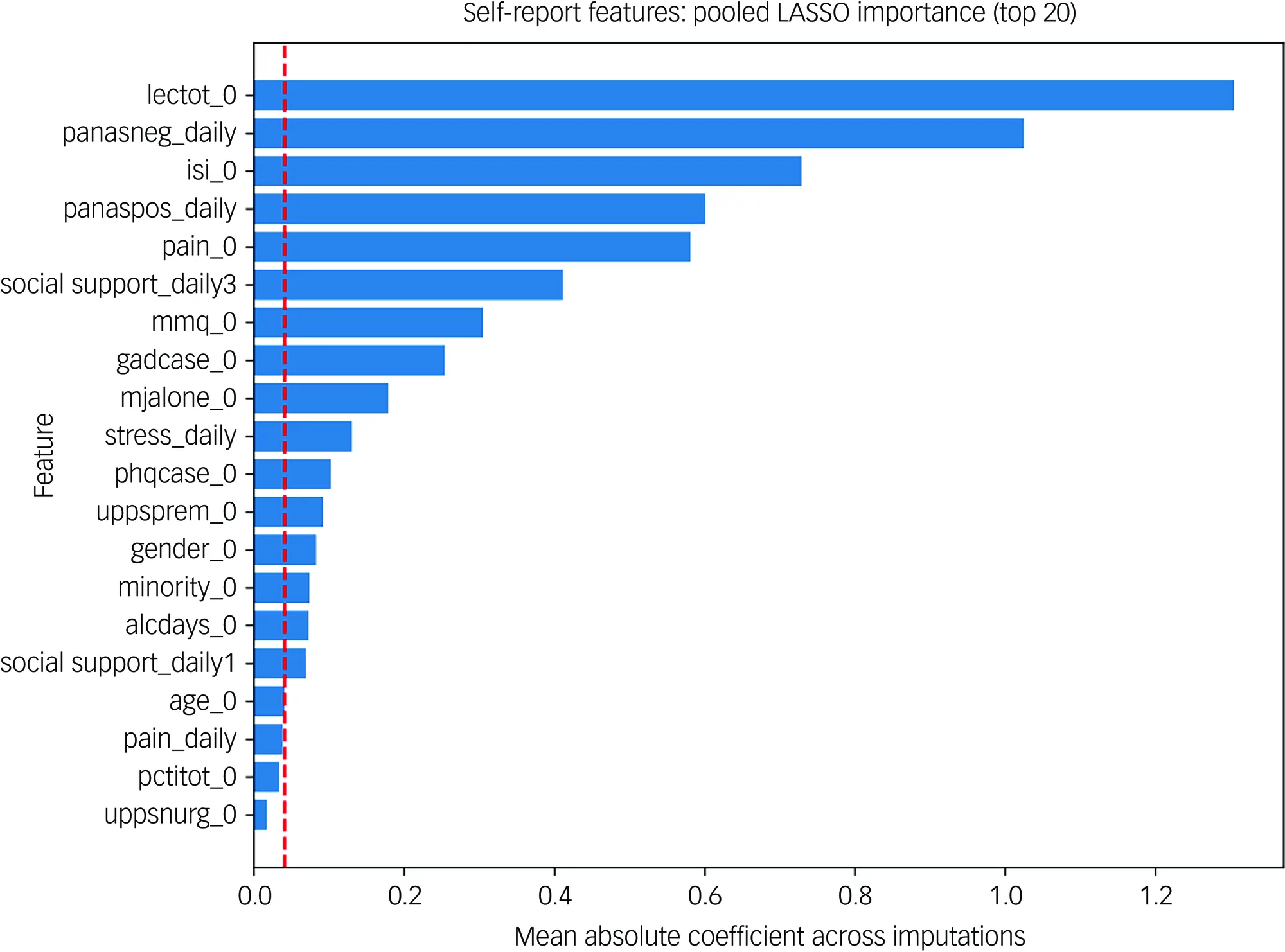
Ranked self-report predictors based on pooled LASSO importance. Values represent the mean absolute coefficient across imputed data-sets. The dashed red line indicates the reference threshold used for visual comparison. Feature labels correspond to self-report predictors included in the model. Where raw variable names are retained, the suffix ‘0’ denotes a baseline measure and ‘daily’ denotes a daily self-report measure. Daily social support item 1 (‘social support_daily1’) and item 3 (‘social support_daily3’) refer to item-level daily social support variables. LASSO, least absolute shrinkage and selection operator; LEC total, Life Events Checklist total score (daily negative affect and daily positive affect were derived from the Positive and Negative Affect Schedule); PANAS, Positive and Negative Affect Schedule; ISI, Insomnia Severity Index; MMQ, Marijuana Motives Questionnaire; GAD caseness, Generalised Anxiety Disorder caseness; PHQ caseness, Patient Health Questionnaire caseness; UPPS-P, Urgency, Premeditation, Perseverance, Sensation Seeking, Positive Urgency Impulsive Behaviour Scale (including premeditation and negative urgency subscales); PTCI, Post-Traumatic Cognitions Inventory.

At the optimal iteration (*k* = 17), the feature set included a combination of self-report and wearable-derived variables. Self-report features comprised baseline PTSD severity, insomnia, depressive and anxiety caseness, daily affect (positive and negative), perceived stress, pain, impulsivity, social support and cannabis-related motives. Demographic and behavioural indicators, such as age, gender, minority status, alcohol use and time spent alone during service, were also retained (see Fig. 1).

Wearable features were drawn from across domains. Activity measures reflected instability in sedentary, lightly active and moderate–vigorous minutes. Distance features captured deviations in daily median and maximum distance. Body composition features included variability in body mass index and body fat. Heart-related predictors centred on deviations in heart rate variability (mean, maximum and median). Sleep features highlighted changes in sleep latency, wake after sleep onset and rapid eye movement sleep duration; step features captured irregularities in step counts and stair climbing (see Supplementary Appendix 2 for rank-order visualisation for each wearable domain).

Overall, no single modality dominated prediction; rather, combining subjective and objective data was necessary for optimal performance. Baseline-adjusted instability features were particularly influential, indicating that departures from individual norms in activity, sleep and autonomic regulation were predictive of short-term PTSD symptom increases.

## Discussion

In this exploratory analysis of recently discharged veterans with elevated PTSD symptoms and problematic cannabis use, we examined whether combining wearable sensor features with daily self-report measures could support the identification of short-term PTSD symptom increases. By using individual baseline scores for each participant, we sought to capture within-person deviations in physiology and behaviour that may be more informative than absolute levels alone. Overall, our findings suggest that integrating passive wearable data with active self-report has potential to characterise periods of heightened symptom risk, and may provide a more complete representation of day-to-day change than either modality in isolation. As this work is exploratory and based on a small sample, further research is required to establish generalisability, refine modelling approaches and determine how such methods could be implemented safely and effectively within clinical or support pathways.

We found that no single feature or sensor modality dominated the predictions; instead, a multimodal feature set spanning sleep, activity, heart rate variability and self-reported mood/stress provided optimal discrimination. Many of the top predictors reflected changes from an individual’s own baseline, underlining the value of baseline-adjusted features in identifying when someone deviates from their normative state. Together, these findings support the feasibility of digital phenotyping for PTSD, where continuous passive data from wearables, combined with active daily assessments, can offer a timely indicator of symptom escalation risk in high-risk populations.

Our results align with and extend prior work on early PTSD detection with machine learning. Traditional PTSD screening relies on periodic questionnaires and interviews, which can miss short-term fluctuations.^19^ Recent studies have increasingly explored objective, technology-driven approaches to monitor PTSD in real time.^35^ For example, Pellegrin and colleagues used physiological responses in a controlled virtual reality stress test to classify PTSD status with roughly 0.8 accuracy.^17^ This underscores that passively collected signals can contain a differentiating signal for PTSD. Similarly, other machine-learning models leveraging multimodal inputs have achieved around 83% accuracy in detecting PTSD.^17^ Our approach achieved comparable classification performance, but in a less controlled, fully ambulatory context. We monitored individuals in their daily lives over the duration of the study period. This real-world context introduces more noise and variability, yet our approach performance suggests that meaningful patterns can still be distilled from the data.

Our findings reinforce known correlates of PTSD and demonstrate their utility in prediction. Sleep disturbances are a hallmark of PTSD,^17^ and a recent meta-analysis confirmed that insomnia and fragmented sleep are highly prevalent in those with PTSD (approximately 63% experience insomnia) and moderately correlated with PTSD severity (aggregate effect size of approximately 0.52).^14^ Consistent with this, our models heavily relied on sleep-related features. For instance, increases in wake after sleep onset and changes in rapid eye movement sleep duration were among top predictors, reflecting the close link between nocturnal arousal and PTSD symptoms. These results are in line with evidence that patients with PTSD have more disrupted sleep continuity and a high co-occurrence of insomnia.^14^

Including self-report features improved model performance, highlighting the value of subjective data for capturing symptoms and context that may not be fully observable through passive sensing alone. Prior analyses from the MAVERICK project showed that daily PTSD symptoms were strongly correlated with negative affect and perceived stress,^8^ and that poorer sleep quality was closely linked to higher stress in this cohort, with stress mediating the association between sleep and next-day substance use.^8^ Our detection approach builds on these findings by incorporating these same domains (sleep-related indicators, perceived stress and affect) alongside wearable-derived features to identify periods of heightened risk for short-term symptom escalation. In doing so, the model reflects the day-to-day coupling of sleep, mood and PTSD symptoms in everyday life. Taken together, these results suggest that integrating active self-report with passive sensor signals provides a more complete representation of risk than either modality alone, combining lived experience with objective behavioural and physiological change.

This study applied baseline-adjusted wearable metrics alongside daily self-reports to characterise short-term PTSD symptom change in a veteran sample. Using each participant’s first 14 days to establish an individual baseline helps account for substantial between-person variability in wearable signals, and supports a focus on deviations from an individual’s typical range. This within-person approach may be more sensitive to clinically meaningful change than relying on absolute, population-normed thresholds, and aligns with broader principles of personalised monitoring.

A further strength is the multimodal nature of the data. PTSD affects physiology, behaviour and emotion, and incorporating features across domains (e.g. sleep, activity, cardiac and respiratory indicators, and self-reported affect and stress) provides a more complete representation of day-to-day functioning than any single modality alone. From an applied perspective, continuous passive monitoring has potential to support earlier identification of periods of increased symptom burden. If integrated carefully into real-world pathways (e.g. within digital health programmes or veteran support services), such approaches could help prompt timely check-ins or additional assessment when patterns suggest rising symptoms, as well as recognise the importance of appropriate thresholds, user autonomy and minimising unnecessary alerts.

The sample was intentionally drawn from recently discharged veterans with elevated PTSD symptoms and problematic cannabis use. This focus is a strength in that it addresses a clinically important subgroup for whom symptom fluctuation, sleep disturbance, affective distress and substance use may be closely linked. However, we recognise it also limits generalisability. Cannabis use may be associated with greater baseline PTSD symptom severity and may influence several of the behavioural and physiological markers analysed, including sleep, activity patterns, affect and autonomic regulation. As such, the feature profile identified in this study may partly reflect the characteristics of this comorbid subgroup rather than PTSD symptom escalation more broadly.

Further, in a clinical setting, we envisage this type of approach being used as a decision-support tool rather than as a diagnostic system. For example, baseline-referenced changes in sleep, activity and self-reported affect could be used to identify periods when a veteran may benefit from a brief check-in, additional self-management support or signposting to clinical or peer-support services. Such outputs would need to be presented to clinicians and users in an interpretable way, with clear thresholds for action and safeguards to avoid over-alerting, inappropriate escalation or replacing clinical judgement. Any future implementation would also need to consider and prioritise consent, privacy, transparency and evaluation of acceptability within real-world care pathways.

Despite our results, this study has several limitations. First, the sample size (74 participants) was small, drawn from a specific subpopulation of recently discharged USA veterans who had elevated PTSD symptoms and concurrent cannabis use. This limits generalisability, and the results may not directly apply to other groups, particularly veterans from other eras or those who are less comfortable with technology. The homogeneity of having all participants with some PTSD symptoms also means we were detecting symptom exacerbations rather than distinguishing completely asymptomatic individuals from PTSD cases. Future work should evaluate performance in broader samples, including those with no PTSD history, to truly test sensitivity and specificity in a screening context.

Second, our outcome measure was based on self-reported PTSD symptom scores (from daily diary entries) rather than a clinical diagnostic assessment. We defined a ‘probable PTSD increase’ as a within-person spike of ≥1 s.d. above baseline, a conservative threshold compared with other studies measuring meaningful symptom change,^36^ but still one that may not capture all clinically significant changes. It is possible that some participants had consistently high PTSD scores that never fluctuated enough to meet the increase criterion (thus appearing as false negatives to the model despite high chronic symptoms). Conversely, others might have transient spikes that triggered the outcome, but did not reflect a lasting clinical deterioration. Relying on self-report also opens the door to reporting bias; however, the use of ecological momentary assessment likely reduced recall error compared to retrospective surveys. Finally, all participants had problematic cannabis use alongside elevated PTSD symptoms. This means the findings should not be assumed to generalise to the wider PTSD population. Cannabis use may be associated with greater baseline symptom severity and may also influence several of the behavioural and physiological signals analysed here, including sleep, activity patterns, affective state and autonomic regulation. As a result, the detected feature profile may partly reflect the specific clinical and behavioural characteristics of this comorbid subgroup. Future studies should validate these models in broader PTSD samples, including individuals without problematic cannabis use, and should examine cannabis use frequency, timing and motives as potential moderators of wearable-derived predictors.

This study provides initial evidence that baseline-referenced wearable sensor data, combined with daily self-reports, may help identify short-term increases in PTSD symptoms among recently discharged veterans with elevated PTSD symptoms and problematic cannabis use. Further validation is required before extending these findings to broader PTSD populations. By using continuous monitoring from consumer devices, the approach highlights the potential relevance of wearable-derived indicators related to sleep continuity, physical activity patterns and autonomic regulation for recognising periods of heightened symptom burden. Although further work is needed to validate these findings and assess their generalisability, this line of research suggests that passive monitoring could complement existing approaches to PTSD care by supporting earlier recognition of symptom escalation between clinical contacts. If integrated appropriately within care and support pathways, such models may enable timely check-ins or stepped support while reducing reliance on infrequent assessments alone. Any future implementation will need to prioritise privacy, transparency and user autonomy, and evaluate acceptability and clinical utility in real-world settings.

## Supporting information

10.1192/bjo.2026.12055.sm001 Leightley et al. supplementary material Leightley et al. supplementary material

## Data Availability

The data used in this study were collected as part of the MAVERICK feasibility trial. The analysis code and additional approaches and analysis are available at https://github.com/DrDanL/maverick-ml-ptsd/tree/main/results.

## Supplementary material

The supplementary material is available online at https://doi.org/10.1192/bjo.2026.12055

## Table 1 Long description

A table with 12 rows and 3 columns summarizing baseline demographics and substance use statistics. The columns are labeled Demographic, Mean, and s.d. / n (%). The table includes data on age, gender, race/ethnicity, number of deployments, employment status, post-traumatic stress disorder, depression, and substance use. Each row provides specific values for these categories. For example, the mean age is 34.2 years with a standard deviation of 8.01, and 18.9 percent of the participants are female. The table also includes data on the average number of deployments, employment status distribution, and mean scores for PTSD and depression. Substance use data includes the average days of cannabis and alcohol use per month, binge-drinking days, and the percentage of participants using study-provided wearable devices and owning Apple smartphones.

Navigate back to Table 1.

## Table 2 Long description

A table comparing the performance of various classifiers using metrics like PR AUC, ROC AUC, Precision, Recall, and F1. The table has 17 rows and 6 columns. The columns are labeled as Classifier, PR AUC (s.d.), ROC AUC (s.d.), Precision (s.d.), Recall (s.d.), and F1 (s.d.). The classifiers listed include LightGBM, XGBoost, Random Forest, CatBoost, Ridge Classifier, Support Vector Machine Linear, AdaBoost, Logistic Regression, Support Vector Machine RBF, Gradient Boosting, k-Nearest Neighbours, Decision Tree, Stochastic Gradient Descent Classifier, ExtraTrees, Decision Tree, and Naive Bayes. Each row provides the performance metrics for the respective classifier.

Navigate back to Table 2.

## Table 3 Long description

A table comparing performance metrics across three iterations. The table has four columns: Iteration, PR AUC (s.d.), ROC AUC (s.d.), Precision (s.d.), Recall (s.d.), and F1 (s.d.). It has three rows of data. Row 1: Iteration, 16; PR AUC (s.d.), 0.64 (0.27); ROC AUC (s.d.), 0.77 (0.23); Precision (s.d.), 0.67 (0.09); Recall (s.d.), 0.63 (0.11); F1 (s.d.), 0.64 (0.10). Row 2: Iteration, 17; PR AUC (s.d.), 0.67 (0.06); ROC AUC (s.d.), 0.78 (0.06); Precision (s.d.), 0.58 (0.08); Recall (s.d.), 0.56 (0.07); F1 (s.d.), 0.57 (0.07). Row 3: Iteration, 18; PR AUC (s.d.), 0.61 (0.29); ROC AUC (s.d.), 0.75 (0.24); Precision (s.d.), 0.27 (0.29); Recall (s.d.), 0.34 (0.32); F1 (s.d.), 0.27 (0.27).

Navigate back to Table 3.

## Table 4 Long description

A table comparing performance metrics of various classifiers. The table has 15 rows and 6 columns. The columns are labeled PR AUC (s.d.), ROC AUC (s.d.), Precision (s.d.), Recall (s.d.), and F1 (s.d.). The rows are labeled with different classifiers: LightGBM, Support Vector Machine linear, Logistic regression, Random Forest, Ridge Classifier, CatBoost, AdaBoost, XGBoost, Support Vector Machine radial basis function kernel, Gradient Boosting, Stochastic Gradient Descent Classifier, ExtraTrees, k-Nearest Neighbours, Decision Tree, and Naive Bayes. Each row contains values for the respective classifier's performance metrics.

Navigate back to Table 4.
