# Supplementary material for: Detecting short-term post-traumatic stress disorder symptom increases among veterans: machine-learning analysis integrating wearable sensor and daily self-report data
Source: BJPsych Open. 2026 Sep 1;12(5):e223. doi: 10.1192/bjo.2026.12055 (PMC13748685; doi:10.1192/bjo.2026.12055)
Supplement: Supplementary file 1 [file S2056472426120559sup001.docx]

**Appendix 1**

This section provides a detailed categorization of the data files used in the study, segmented into various health and activity-related domains. This categorization is based on data similarity and sensor modality.

1. Distance Files

The following files contain data on distances covered:

distance

exercise-distance

activities-distance

2. Steps Files

The following files record step counts:

steps

exercise-steps

activities-steps

3. Activity Files

The following files contain various activity-related data:

activities-floors

activities-calories_burned

activities-activity_calories

sedentary_minutes_activity_value

lightly_active_minutes_activity_value

moderately-fairly_active_minutes_activity-value

very_active_minutes_activity-value

activity

4. Sleep Files

The following files record sleep-related data:

sleep-minutes-asleep

sleep-timein-bed

sleep-duration

sleep-main-sleep

sleep-type

sleep-info-code

sleep-log-type

sleep-asleep-count

sleep-minutes-awake

sleep-awake-count

sleep-wake-count

sleep-wake-minutes

sleep-wake-thirtyday-avgminutes

sleep-light-minutes

sleep-light-count

sleep-light-thirdtyday-avgminutes

sleep-deep-count

sleep-deep-thirtyday-avgminutes

sleep-rem-minutes

sleep-rem-count

sleep-rem-thirtyday-avgminutes

sleep-deep-minutes

sleep-minutes-tosleep

sleep-minutes-afterwake

sleep-efficiency

sleep-restless-count

sleep-restless-minutes

5. Exercise Files

The following files contain exercise-related data:

exercise-activity_name

exercise-activity_typeid

exercise-duration

exercise-activeDuration

exercise-logType

exercise-hasGps

exercise-should_fetchdetails

exercise-has_active_zone_minutes

exercise-source_type

exercise-source_id

exercise-source_urls

exercise-source_tracker_features

exercise-speed

exercise-pace

exercise-average_heart_rate

exercise-source_name

exercise-calories

exercise-manual_values_specified_calories

exercise-swim_lengths

exercise-pool_length

exercise-pool_length_unit

apple_exercise_time

6. Foods Files

The following files record food intake data:

foods-Calories_In

foods-Fat_g

foods-Fiber_g

foods-Carbs_g

foods-Sodium_mg

foods-Protein_g

foods-Water_fl_oz

foods-Meal

foods-Food

foods-Food_Calories

7. Heart Rate Files

The following files contain heart rate-related data:

resting_heart_rate_value_error

activeZoneMinutes_totalMinutes

time_in_heart_rate_zones-value-valuesInZones-IN_DEFAULT_ZONE_1

time_in_heart_rate_zones-value-valuesInZones-IN_DEFAULT_ZONE_2

time_in_heart_rate_zones-value-valuesInZones-IN_DEFAULT_ZONE_3

Heart Rate Variability Details-low_frequency

Heart Rate Variability Details-high_frequency

Heart Rate Variability Details-rmssd

Heart Rate Variability Details-coverage

Daily Heart Rate Variability Summary-rmssd

Daily Heart Rate Variability Summary-nremhr

Daily Heart Rate Variability Summary-entropy

heart_rate_variability

8. Respiratory Rate Files

The following files contain respiratory rate data:

respiratory_rate

Daily Respiratory Rate Summary-daily_respiratory_rate

Respiratory Rate Summary-full_sleep_breathing_rate

Respiratory Rate Summary-full_sleep_standard_deviation

Respiratory Rate Summary-full_sleep_signal_to_noise

Respiratory Rate Summary-deep_sleep_breathing_rate

Respiratory Rate Summary-deep_sleep_standard_deviation

Respiratory Rate Summary-deep_sleep_signal_to_noise

Respiratory Rate Summary-light_sleep_breathing_rate

Respiratory Rate Summary-light_sleep_standard_deviation

Respiratory Rate Summary-light_sleep_signal_to_noise

Respiratory Rate Summary-rem_sleep_breathing_rate

Respiratory Rate Summary-rem_sleep_standard_deviation

Respiratory Rate Summary-rem_sleep_signal_to_noise

9. SpO2 Files

The following files record blood oxygen saturation data:

Daily SpO2-average_value

Daily SpO2-lower_bound

Daily SpO2-upper_bound

Minute SpO2-value

10. Demographic VO2 Files

The following files contain VO2 max data:

demographic_vo2_max-value-demographicVO2Max

demographic_vo2_max-value-demographicVO2MaxError

demographic_vo2_max-value-filteredDemographicVO2Max

demographic_vo2_max-value-filteredDemographicVO2MaxError

11. Device Temperature Files

The following files record device temperature data:

Device Temperature-temperature

Device Temperature-sensor_type

12. Body Files

The following files contain body composition data:

body_bmi

body_Fat

**Appendix 2**
